# Explanation of the Formation of Complexes between Representatives of Oxazolidinones and HDAS-β-CD Using Molecular Modeling as a Complementary Technique to cEKC and NMR

**DOI:** 10.3390/ijms22137139

**Published:** 2021-07-01

**Authors:** Wojciech Bocian, Elżbieta Bednarek, Katarzyna Michalska

**Affiliations:** 1 Falsified Medicines and Medical Devices Department, National Medicines Institute, Chełmska 30/34, 00-725 Warsaw, Poland; w.bocian@nil.gov.pl (W.B.); e.bednarek@nil.gov.pl (E.B.); 2 Department of Synthetic Drugs, National Medicines Institute, Chełmska 30/34, 00-725 Warsaw, Poland

**Keywords:** chiral cEKC, molecular modeling, non-covalent interactions, NMR, oxazolidinone

## Abstract

Molecular modeling (MM) results for tedizolid and radezolid with heptakis-(2,3-diacetyl-6-sulfo)-β-cyclodextrin (HDAS-β-CD) are presented and compared with the results previously obtained for linezolid and sutezolid. The mechanism of interaction of chiral oxazolidinone ligands belonging to a new class of antibacterial agents, such as linezolid, tedizolid, radezolid, and sutezolid, with HDAS-β-CD based on capillary electrokinetic chromatography (cEKC), nuclear magnetic resonance (NMR) spectroscopy, and MM methods was described. Principles of chiral separation of oxazolidinone analogues using charged single isomer derivatives of cyclodextrin by the cEKC method were presented, including the selection of the optimal chiral selector and separation conditions, complex stoichiometry, and binding constants, which provided a comprehensive basis for MM studies. In turn, NMR provided, where possible, direct information on the geometry of the inclusion complexes and also provided the necessary structural information to validate the MM calculations. Consequently, MM contributed to the understanding of the structure of diastereomeric complexes, the thermodynamics of complexation, and the visualization of their structures. The most probable mean geometries of the studied supramolecular complexes and their dynamics (geometry changes over time) were determined by molecular dynamics methods. Oxazolidinone ligands have been shown to complex mainly the inner part of cyclodextrin, while the external binding is less privileged, which is consistent with the conclusions of the NMR studies. Enthalpy values of binding of complexes were calculated using long-term molecular dynamics in explicit water as well as using molecular mechanics, the Poisson–Boltzmann or generalized Born, and surface area continuum solvation (MM/PBSA and MM/GBSA) methods. Computational methods predicted the effect of changes in pH and composition of the solution on the strength and complexation process, and it adapted the conditions selected as optimal during the cEKC study. By changing the dielectric constant in the MM/PBSA and MM/GBSA calculations, the effect of changing the solution to methanol/acetonitrile was investigated. A fairly successful attempt was made to predict the chiral separation of the oxazolidinones using the modified cyclodextrin by computational methods.

## 1. Introduction

Due to the wide scope of the scientific purpose of this study, it was necessary to systematize and clearly present the results obtained so far; hence, the introduction consists of the following parts: Section 1.1 is devoted to the chemical characteristics of antimicrobial oxazolidinone analogues and the possibility of using nuclear magnetic resonance (NMR) spectroscopy and molecular modeling (MM) to explain the structure of supramolecular oxazolidinone complexes. Section 1.2 presents the evolution of views on chiral recognition mechanisms and characterizes cyclodextrins and their potential for the formation of inclusion complexes and intermolecular interactions with chiral selector and selectand. Section 1.3 presents the results of interactions between the tested oxazolidinones and the selected chiral selector, heptakis-(2,3-diacetyl-6-sulfo)-β-cyclodextrin (HDAS-β-CD) by the cEKC and NMR methods.

### 1.1. General Concept

**Figure 1:**
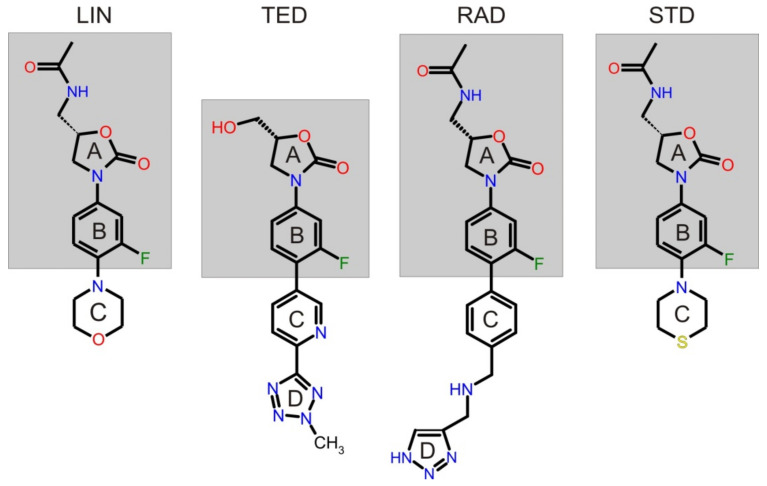
Molecular structures of linezolid (LIN), tedizolid (TED), radezolid (RAD), and sutezolid (STD). The LIN, TED, RAD, and STD precursors are marked in the frame.

Oxazolidinones [1] are chiral, synthetic compounds active against Gram-positive bacteria and belong to the most-expected class of antimicrobial agents due to the problems associated with the rapid spread of antibiotic-resistant pathogenic bacteria. There are two FDA/EMA (Food and Drug Administration, European Medicines Agency) approved oxazolidinones, linezolid (LIN) and tedizolid (TED), as well as two that are still in clinical trials, radezolid (RAD) and sutezolid (STD). The molecular structures of LIN N-({(5*S*)-3-[3-fluoro-4-(morpholin-4-yl)phenyl]-2-oxo-oxazolidin-5-yl}methyl) acetamide, TED (5*R*)-3-{3-fluoro-4-[6-(2-methyl-2H-1,2,3,4-tetrazol-5-yl)pyridin-3-yl]phenyl}-5-(hydroxymethyl)-1,3-oxazolidin-2-one, RAD N-{[(5*S*)-3-[3-fluoro-4-(4-{[(1H-1,2,3-triazol-5-ylmethyl)amino]methyl}phenyl)phenyl]-2-oxo-1,3-oxazolidin-5-yl]methyl}acetamide, and STD *N*-({(5*S*)-3-[3-fluoro-4-(thiomorpholin-4-yl)phenyl]-2-oxo-oxazolidin-5-yl}methyl) acetamide are shown in Figure 1. The single isomers of the tested oxazolidinones have one chiral center at position C5 of the oxazolidinone ring substituted with a methyl acetamide group, as in the case of (5*S*)-LIN, (5*S*)-RAD, and (5*S*)-STD, or a hydroxymethyl substituent in (5*R*)-TED, which is associated with the antimicrobial drug activity [1]. However, (5*S*)-LIN, (5*S*)-RAD, (5*S*)-STD, and (5*R*)-TED have the same spatially oriented substitution at the C5 position of the oxazolidinone ring.

LIN was approved by the FDA on April 2000. Fourteen years later, in June 2014, TED was registered by the FDA, and in January 2015, it was approved by the EMA. RAD has completed two Phase 2 clinical trials (http://clinicaltrials.gov, accessed on 12 July 2021), the first in community-acquired pneumonia (CAP, NCT00640926) and the second in uncomplicated skin and skin-structure infections (SSSI, NCT00646958), but it still remains at the clinical development stage. The last of this group, STD, is currently recruiting to Phase 4 development (NCT03237182) and Phase IIB (NCT03959566) in adults with newly diagnosed, uncomplicated, smear positive, and drug-sensitive pulmonary tuberculosis, and the most recently completed Phase 2 clinical trials [2] in patients with drug-sensitive pulmonary tuberculosis (NCT01225640). It is also worth noting that other oxazolidinones are also in the early stages of clinical trials: delpazolid (LCB01-0371, LegoChem Bioscences Inc.) with cyclic amidrazone and contezolid acefosamil (MRX-I/MRX-4, MicuRx Pharmaceuticals Inc.) completed Phase 2 (NCT03747497) in adult patients with acute bacterial skin and skin structure infection [3].

Our group has been conducting research on the enantiomeric separation of oxazolidinones (LIN, TED, RAD, STD) by capillary electrokinetic chromatography (hereinafter referred to as cEKC) for several years. NMR and MM as complementary methods to cEKC were used to elucidate and understand the interaction between oxazolidinones and the selected chiral selector, HDAS-β-CD. The results obtained for LIN [4,5], TED [6,7,8], RAD [9,10,11], and STD [12] have already been published by our research group.

Principles of chiral separations of the oxazolidinones analogues (indicated above) by using charged single isomer derivatives of cyclodextrin (CD) by cEKC were performed [4,6,9,12]. As part of the optimization of enantioseparation by the cEKC method, the most versatile chiral selector was selected among the tested, and the separation conditions were optimized.

Taking into account the chemical nature of the tested oxazolidinones, it can be concluded that: (i) LIN is unionized at buffer above pH 3, while at low pH, it can be considered a weak monobasic compound due to the protonation of the nitrogen atom on the morpholine ring, whereas (ii) TED, based on pKa calculations performed by Chemicalize in the pH range 2–12, exists as a non-charged analyte, (iii) RAD contains a biphenyl ring system linked by an aminomethyl bridge to a triazole ring, which increases the ionization and hydrophilicity of the molecule and gives it a dibasic character (below pH 7.0), and (iv) STD contains a thiomorpholine ring instead of a morpholine ring, which constitutes the difference from its predecessor, LIN, which makes STD a weak monobasic compound, but stronger than LIN, due to the protonation of the nitrogen atom on the thiomorpholine ring. Thus, in cEKC experiments, anionic CDs were tested, from hydrophilic anionic single isomer to moderately hydrophobic and hydrophobic CDs as carriers of uncharged compounds, to obtain differences in electrophoretic velocity during migration. It was also assumed that in the case of protonated forms of LIN, RAD, and STD at low pH of background electrolyte (BGE) with negatively charged CDs, electrostatic interactions will occur. The cEKC results [4,6,9,12] indicated that moderately hydrophobic HDAS-β-CD is the most selective chiral selector concerning the new derivatives of oxazolidinone, which facilitates their enantiomeric resolution both under aqueous (ACE) and non-aqueous capillary electrophoresis (NACE). At ACE, HDAS-β-CD differentiate the electrophoretic mobility of enantiomers, both LIN [4], RAD [9], and TED, although in the case of TED, acetonitrile (ACN) was required [6], but also for precursors of LIN/RAD and precursors of TED [6]. LIN/RAD precursors consist of A (oxazolidinone) and B (phenyl) rings only, but they do not have C, (morpholine) or pyridine (C) and triazol (D) rings for LIN and TED, respectively. In contrast, TED precursors, similar to LIN/RAD precursors, only have an A ring; however, in this case, they have a hydroxymethyl substituent at the C5 position of the oxazolidinone ring and a B ring but do not contain C and D rings (Figure 1).

Additionally, HDAS-β-CD provided the baseline separation of STD enantiomers at NACE [12]. However, under NACE conditions, hydrophobic heptakis-(2,3-dimethyl-6-sulfo)-β-cyclodextrin (HDMS-β-CD), and not HDAS-β-CD, proved to be the most versatile chiral selector against the tested oxazolidinones because it separated both LIN and STD, while HDAS-β-CD separated the STD enantiomers exclusively.

As cEKC suffers from the disadvantage of not being able to provide any direct structural information about complex formation, NMR spectroscopy and MM were used. First of all, it was expected that the existence of an interaction between the oxazolidinones (LIN, TED, RAD, or STD) and HDAS-β-CD could be proven based on the NMR study, and whether the formation of inclusion complexes or external interactions was dominant could be determined. Various NMR techniques can be used to obtain information on complex structure, stoichiometry, as well as to estimate host–guest binding affinity.

On the basis of the analysis of chemical shifts, changes of the guest proton signals, as well as information from Nuclear Overhauser Effect Spectroscopy (NOESY) or Rotating-frame Overhauser Enhancement Spectroscopy (ROESY) experiments, one can conclude how the depth and which part of the guest molecule penetrates into the CD cavity. It should be emphasized that for research in solution, 2D NMR experiments, NOESY, as well as ROESY are some of the few experimental methods that can be used to obtain information about the penetration of the CD cavity by the guest. Complex stoichiometry is usually obtained via the analyses of the standard Job plot. The binding constants of the CD complexes can be estimated by using a pulsed field gradient spin-echo NMR PFGSE experiment or NMR titration method.

In our study, NMR measurements were performed in similar solutions (solvent, ionic strength, pH) in which the enantiomeric separation was obtained by the cEKC method. Unfortunately, the conditions chosen as optimal for the cEKC separation were generally not appropriate for NMR measurements. Particularly, the low solubility of the tested compounds in selected solvents as well as the broadening of the guest signals after the addition of HDAS-β-CD meant that not all of the planned NMR measurements could be carried out, and some of the results could not be interpreted. For these reasons, NMR measurements were only performed for LIN for both the biological active isomer (*5S*) and the inactive (*5R*) [5], whereas in the case of TED and STD, NMR studies were limited to isomers exhibiting antimicrobial activity, (*5R*)-TED [8] and (*5S*)-STD [12].

MM methods were used as complementary techniques to the results obtained by cEKC and NMR to elucidate the enantioseparation mechanism. For LIN [5] and STD [12], the MM results along with the cEKC results have already been published by our research group. However, to get the most complete overview of the results obtained for the entire oxazolidinone group, it was decided to compare and discuss the results obtained for TED and RAD with those already published for LIN and STD. This seems important for comparing and positioning individual representatives of oxazolidinone in terms of the strength of their interaction with HDAS-β-CD.

The aim of this article is to explain and understand the process of complex formation between ligands of oxazolidinones and HDAS-β-CD using MM as a complementary technique to cEKC and NMR, which may contribute to the advancement of our knowledge about non-covalent intermolecular interactions as well as the supramolecular chemistry of the novel analogues of oxazolidinones.

### 1.2. Evolution of Views on the Mechanisms of Chiral Recognition

Many review papers and special issues devoted entirely to the problem of enantioseparations provide a historical overview of chiral recognition processes, highlight the significance of these research, and summarize the most important theoretical principles [13,14,15,16,17,18,19,20,21,22,23]. These papers cover issues ranging from the “three-point attachment model” through discussion of the different thermodynamic aspects in relation to chiral selector–selectand interactions and fundamental aspects of enantioseparations, to computational techniques that are also discussed as a theoretical complement to experimental chiral separations of liquid chromatography and cEKC. Recently, Chankvetadze [20] presented an overview of the formation and development of the contemporary theory of enantioseparation in the cEKC. Bernardo-Bermejo et al. [23] presented the most recent technological and methodological achievements in capillary electrophoresis (CE), while Yu and Quirino [21] focused on new trends in chiral selectors in this field.

CDs [24,25] along with crown ethers, cyclic polyamines, calixarenes, cucurbiturils, and pillar[n]arenes, are mostly found in inclusion complexes. The most commonly used CDs are constructed of six (α-CD), seven (β-CD), or eight (γ-CD) D-glucose units linked by α-1,4 glycosidic bonds. CDs have a wreath-shaped truncated cone that is open at both ends. One of the CD cones is surrounded by secondary hydroxyl groups at the C2 and C3 positions, while the narrower CD cone is surrounded by more polar, primary hydroxyl groups located at the C6 position. In the case of HDAS-β-CD, the hydroxyl groups at the C2 and C3 positions are substituted with moderately hydrophobic acetyl groups, while in the C6 position, the primary groups are substituted with charged sulfate groups. The conical cylinder structure of these cyclic oligosaccharides contains a cavity lined with H3/H5 protons, and ion–electron pairs from the glycosidic oxygen bridges produce a high electron density, thus creating a hydrophobic character and a slightly Lewis-base character of the interior. Meanwhile, the primary hydroxyl groups in the C6 position and secondary hydroxyl groups in the C2 and C3 positions give the surface of the CD hydrophilic character. Due to the C1-O-C4 glycosidic bridges that connect the individual glucopyranose units, the α-CD and γ-CD molecules are flexible. However, native β-CD is a fairly rigid structure due to the odd number of glucopyranose units that form the secondary belt due to the interaction of the C2 hydroxyl group of one glucopyranose unit with the C3 hydroxyl group of the adjacent glucopyranose unit, resulting in low solubility in water [26]. In this context, the chemical modification of native β-CD disrupts the hydrogen bonds belt and, thus, improves water solubility.

CDs have the ability to create host–guest inclusion complexes precisely because of the presence of a hydrophobic interior and some flexibility, thus forming a host molecule for various molecules or ions. The geometry of the guest determines the selectivity of the complexing process. One of the conditions for the formation of an inclusion complex is that the guest molecules have to penetrate into the CD completely or at least to a large extent. However, spatial compatibility alone is insufficient for chiral differentiation. It should be complemented by the interaction of structural elements of the guest with functional groups present on the rim of the toroidal structure of the CD [27]. Interaction between substituents on the asymmetric center of the analyte and the hydroxyl groups or other substituted groups at positions C2, C3, and C6 determine the chiral differentiation. The inclusion complex is usually more stable than the substrates, and thus, the system is more favorable energetically. Generally, the molar ratio of complex molecules of the guest to the host is 1:1; however, 2:1, 1:2, 2:2, or even more complicated associations or higher-order equilibria exist. In most cases, the complexation process is regioselective and stereospecific, and the CDs show substrate selectivity. In addition to the formation of inclusion complexes [5,28,29], it is also possible to create a steroselective external interaction [30,31]. This image becomes complicated if the chiral differentiation is also co-decided by the achiral factors, e.g., organic modifiers or components of the BGE [32]. The use of NMR spectroscopy to better understand the mechanisms of enantiomer separation in cEKC has been exhaustively discussed by Salgado and Chankvetadze [33], primarily giving insight, at the molecular level, into the enantioselective non-covalent intermolecular interactions between host and guest, leading to cEKC enantioseparation. The review [33] is an attempt to illustrate and indicate the main difference between the chiral separation in cEKC, while emphasizing the uniqueness of CE and chromatography in the example of reversing the enantiomer migration order (EMO), which in CE can be adjusted by modifying the experimental parameters without the need to change the affinity pattern of the enantiomers toward the chiral selector, while in chromatographic techniques (excluding that with a chiral mobile phase additive), CE can be adjusted mostly by changing the absolute stereochemical configuration of the chiral selector. Moreover, as argued by Chankvetadze [20], the most striking difference between chromatographic and electrophoretic enantioseparation is that cEKC enables enantioseparation to be obtained even when the binding constants of both enantiomers to the chiral selector are equal. Hence, it seems that CE is one of the most sensitive tools for detecting very weak non-covalent enantioselective interactions due to the high separation efficiency, which in turn allows the baseline separation of enantiomers to be observed, even with a very small difference in energy between the formed transient diastereomeric complexes at the level of a few kJ/mol [20].

In CE, the chiral selector is usually added to the BGE; therefore, selector–selectand or host–guest complexes are mobile, so on this basis, two principles governing enantioseparation are possible: (i) the chromatographic enantioselective recognition occurring at the molecular level results from the differences in complexation constants between the enantiomers and the selector, and (ii) the electrophoretic enantioselective separation based on the different mobility of formation of the transient diastereomeric complexes between the analytes and selectors in the thermodynamic equilibria [20]. The principle of separation and the enantioseparation mechanisms in cEKC have been extensively described in a recently published review article on the subject [23].

The formation of transient diastereomeric complexes between the chiral selector and the chiral analytes is accomplished through a variety of interactions, both ionic as well as ion–dipole or dipole–dipole stacking, π–π and hydrophobic interactions, hydrogen and halogen bonding interactions, van der Waals, and dispersion forces. Strong long-range interactions, such as ionic interactions, are often considered to be the first involved in the initial stage; however, since both enantiomers of an ionized solute are capable of forming these interactions, they may not be stereoselective. Hence, both enantiomers are non-stereoselectively bound to the CD. In contrast, short-range interactions such as hydrogen bonds and π–π interactions are primarily responsible for the formation of stereoselective bonds [15,34]. Our research group reached similar conclusions by analyzing the results obtained for RAD and HDAS-β-CD/ HDMS-β-CD under low pH conditions [9].

The hydrophobic effects formation of an inclusion complex are described by Biedermann et al. [35], who, in light of new results obtained with supramolecular complexes, questioned traditional descriptions of the hydrophobic effect on the basis of entropic arguments or the calculation of solvent occupied surfaces. In addition to the formation of intermolecular interactions, the expulsion of high-energy water from the CD cavity may play a role in the formation of chiral complexes. The interior of these molecules is occupied by water molecules in a number of cavity-dependent quantities. These particles are unorganized; they could not form a stable network because only a limited number of hydrogen bonds are possible. As a result, the enthalpy becomes a driving force capable of creating host-type complexes, as it results in more favorable thermodynamic systems. Water molecules are thrown out of the interior of the cavity outside where there is a greater probability of creating more stable hydrogen bonds, and the resulting guest–host is maintained on the basis of hydrophobic and/or van der Waals interactions [36].

### 1.3. Published Results of cEKC Experiments and NMR Measurement of New Oxazolidinone Analogues

Table 1 summarizes the fundamental parameters obtained in the cEKC studies for selected oxazolidinones also in the context of EMO reversal.

The effective mobility of both LIN enantiomers becomes anionic upon complexation with HDAS-β-CD, and both anionic peaks were dragged by the strong electroosmotic flow (EOF) in normal polarity mode to the detector [4]. The more strongly bound enantiomer, (*R*)-LIN, was eluted second at the normal (positive) polarity. In order to reverse the EMO, the type of capillary was changed to neutral, and the polarity of the high-voltage supply was reversed to negative. Reversal of EMO was achieved without the need to change the affinity pattern of the enantiomers toward the chiral selector, which is unique for CE compared to the chromatography technique, as already mentioned above, but it was achieved by suppression of EOF and reverting the polarity of the high-voltage supply. Hence, at cEKC, depending on the mobility effects occurring in the separation system, the more strongly bound enantiomer, in this particular case, (*R*)-LIN, may migrate as the first (at low pH buffer) or as the second peak at high pH values of the BGE. In neutral capillaries with zero EOF flow, the negative HDAS-β-CD is employed as a carrier for the neutral LIN due to self-mobility of the charged CD.

NMR studies were used to obtain information on complex stoichiometry via the analyses of the standard Job plot, while binding constants were estimated by the analysis of the diffusion coefficient of the HDAS-β-CD and (*R*)-LIN or (*S*)-LIN, both at pH 7.0 and pH 2.4 [5]. Based on the chemical shifts changes of signals of HDAS-β-CD protons upon the addition of LIN, it was concluded that both (*S*)- and (*R*)-enantiomers form inclusion complexes with HDAS-β-CD, although the interaction of host–guest not involving the cavity is also possible. In addition, it was shown that for both enantiomers, total inclusion to cavity of HDAS-β-CD takes place (chemical shifts changes, Δδ, for protons H3 and H5 of HDAS-β-CD meet the following relationship: ΔδH3 < ΔδH5). The observed chemical shifts changes of protons signals of LIN can indicate that the inclusion via the morpholine part is equally probable for both enantiomers, whereas inclusion via the oxazolidinone parts is more probable for the (*R*)-enantiomer. For both enantiomers, the 1:1 complex stoichiometry was detected. The binding constants, K_a_, estimated by using the NMR PFGSE experiment, were of the order of 30–80 M^−1^ (Table 1). Similar values of the binding constants for LIN were obtained using the cEKC technique, calculating the constant K_a_ in the LIN and RAD pair [9], where the leading compound in this pair was RAD. (*R*)-LIN is a little more strongly bonded than (*S*)-LIN with HDAS-β-CD both at pH 2.4 and pH 7.0, which coincides well with the EMO observed at pH 7.0 in cEKC using HDAS-β-CD as a chiral selector. In contrast, considering the mobility effect of the cEKC separation system in a low pH buffer, (*R*)-LIN, with a higher bond strength, was the first to migrate.

The second of the tested oxazolidinones, TED, revealed the influence of the achiral factor on enantiomeric differentiation, mainly the effect of organic solvents, which have been extensively studied from amphiprotic to aprotic on the chiral recognition of TED enantiomers [6], while the effect of organic solvents on the chiral resolution of LIN enantiomers was not observed. The addition of a relatively small volume of acetonitrile (ACN) (tested range from 1 to 36% *v*/*v*) to BGE consisting of 37.5 mM HDAS-β-CD in borate buffer and pH 9.0 proved crucial in distinguishing the TED enantiomers, resulting in a partial separation of (*R*)-TED and its (*S*)-enantiomer. The further increase in ACN concentration led to a gradual increase in the resolution of the TED enantiomers. The addition of ACN to the BGE had a greater effect on TED than on the LIN enantiomers because LIN isomers did not require the addition of an organic solvent to achieve enantioseparation. Moreover, it was proven that changing one of the achiral components of the BGE, borate buffer on formate buffer, which is capable of acting as a proton donor, increased the resolution of the TED isomers in HDAS-β-CD at the formic buffer compared to the borate buffer. However, as with HDAS-β-CD in borate buffer, the addition of ACN to the BGE was crucial and had a high impact on the resolution of the TED enantiomers. Apparent average binding constants for LIN and TED isomer pairs at HDAS-β-CD confirmed that in the formate buffer, both TED and LIN create more stable systems (Table 1). The values of the calculated apparent average binding constants were about two and a half times higher under formic buffer (pH 4.0) conditions than under borate buffer (pH 9.0) conditions. However, due to the non-correction of the viscosity and the lack of correction for the ionic strength of the buffer, as well as the low precision of the calculated constants, it is difficult to rely on these results. These doubts are also confirmed by the fact that the values of the calculated K_a_ constants for TED presented in [6] are several times higher than the K_a_ values calculated by the NMR method in [8]. A similar situation occurs in the case of LIN, where the values of the calculated K_a_ constants presented in [6] are at least one order higher than the values of K_a_ calculated by both the NMR method in [5] and the cEKC method in [9]. By analyzing all the input data used to determine the K_a_ including the measurement condition, it can be suspected that in [6], an inappropriate range of chiral selector concentrations was used to calculated the K_a_. An insufficient number of measuring points, or even lack of measuring points especially in the range of 0–20 mM (borate buffer), as well as too long migration times for TED, about 50 min at a concentration of 45 mM (formic buffer), are responsible for the inappropriate assumption and the choice of the inappropriate calculation method. Ultimately, it should be stated that the K_a_ data given in [6] may be subject to considerable error.

According to the conducted literature review on the influence of achiral factors, including the effect of the buffer additives on the structure of analyte–CD complexes, thanks to the results of NMR measurements and MM for the dipeptide Ala-Tyr and β-CD in the presence of urea, such an influence has been confirmed. NMR spectroscopy revealed differences in the complexation of the peptide enantiomers by β-CD in the absence and presence of urea, suggesting the stabilization of the complex by the phenolic hydroxyl group of tyrosine [37]. Similarly, the effect of 1,2-dibromoethane on the complexation of naproxen and β-CD was observed [38].

To mimic the optimal conditions developed at cEKC (HDAS-β-CD dissolved in formic buffer (pH 4.0) with ACN), an attempt was made to measure NMR in similar solutions. However, the results were practically impossible to interpret due to, inter alia, the low solubility of TED. Therefore, our research group decided to perform the systematic NMR studies in aqueous solution mainly for the (*R*)-enantiomer of tedizolid phosphate ((*R)*-TED-PO_4_), which has better water solubility (0.3 mm) than the parent tedizolid ((*R)*-TED-OH) (0.01 mm). The NMR results confirmed that the inclusion complex between (*R)*-TED-PO_4_ and HDAS-β-CD is formed. Based on the chemical shifts changes, Δδ, for protons H3 and H5 of HDAS-β-CD, it was found that there is only a partial inclusion of the (*R*)-TED-PO_4_ into the HDAS-β-CD cavity (ΔδH3 > ΔδH5) as opposed to LIN, where total inclusion takes place. Similar to the LIN complex with HDAS-β-CD, for (*R*)-TED-PO_4_, the 1:1 complex stoichiometry was detected. The mode of complexation was determined from the rotating-frame Overhauser effect (ROE) spectra. The results indicate that (*R*)-TED-PO_4_ crosses the HDAS-β-CD internal cavity, with the pyridine ring pointing toward the narrow rim and the aromatic ring substituted with fluorine located close to the wide rim. This means that the (*R*)-TED-PO_4_ molecule with the triazole ring enters the HDAS-β-CD cavity through the wider rim. The binding constants, *K_a_*, for the HDAS-β-CD/(*R*)-TED-PO_4_ complex estimated by using the NMR PFGSE experiment were of the order of 100 M^−1^ [8].

Additionally, in order to explain the influence of the individual structural elements of the analyzed oxazolidinone analogues, LIN and TED precursors were synthesized [9,10]. The TED precursors did not require the addition of ACN to the BGE to achieve enantioseparation, unlike the TED itself, while the LIN precursors required the presence of ACN to obtain the enantioseparation, also quite unlike LIN. On this basis, we conclude that the C or both C and D rings in the TED molecule have a significant effect on enantioselective interaction with HDAS-β-CD, and that the morpholine ring (C) plays a role in chiral differentiation the LIN isomers [6]. As in the case of the LIN and TED precursors, the analysis of the behavior of RAD precursors carried out under optimal conditions for the separation of RAD enantiomers also indicates the contribution of further rings (C or C and D) on the interaction with HDAS-β-CD [9].

Radezolid is a stronger base than the other tested oxazolidinones; therefore, it interacts more strongly with the chiral selector. It has been proven that in the case of protonation of the nitrogen atom at the RAD molecule (at low pH) in the methylamine link, very strong interactions occur with all of the tested CDs: heptakis-(2,3-dihydroxy-6-sulfo)-β-cyclodextrin (HS-β-CD), HDAS-β-CD, HDMS-β-CD [9].

In any case, at low pH values between protonated RAD and an anionic CD, the interactions are strong but at the same time non-selective (parasitic). Such strong interactions between RAD and HDAS-β-CD in the low pH buffer were most likely due to the inclusion of RAD into the interior of the CD as well as the simultaneous electrostatic attraction. The lack of enantiomeric resolution indicates that the strong non-enantiospecific interactions outweighed the subtle enantiodiscriminatory effect, which prevented the separation of RAD enantiomers. Therefore, in order to be able to discriminate positively charged RAD enantiomers at low pH buffer, it was necessary to reduce the relatively strong electrostatic interactions by increasing the temperature from 17 to 52 °C in the case of HDAS-β-CD [9], which was the opposite to separate the enantiomers of LIN (Table 1). In the case of LIN, lowering the temperature led to a greater resolution between the isomers. To explain the geometry of supramolecular complex formation, taking into account the difficulty of transferring the optimized conditions proposed in cEKC to NMR measurements (due to the large broadening of the bandwidth), electronic circular dichroism (ECD) spectroscopy, as a complementary technique to cEKC, was used. In the ECD study, the conditions developed during cEKC, including the temperature gradient from 10 to 90 °C, were transferred to the ECD. Increasing the temperature led to the weakening of intermolecular interactions, which was confirmed by the decrease in the molar value of ellipticity. RAD spectra from HDMS-β-CD differed from those previously observed for RAD with HDAS-β-CD, indicating the different geometry of the RAD complexation process for the two CDs used. In both cases, inclusion complexes were established [9].

Apparent average binding constants for each enantiomer of RAD and LIN for HDAS-β-CD at pH 2.5 and 6.6 and stoichiometries, which in the case of LIN was 1:1, while for RAD, it was both 1:1 and 1:2 (guest:host), are presented in Table 1 [9].

Concerning STD, due to the low water solubility of STD, NACE became the technique of choice [12]. The advances in chiral separations by NACE in pharmaceutical and biomedical analysis have been extensively described [39].

The overarching goal of our study was to obtain STD and LIN separation, which was necessary in order to directly determine the influence of the structure of the tested compounds on the complexing process and, at the same time, to obtain visual evidence of the relevant protonation of nitrogen in the morpholine or thiomorpholine rings of LIN and STD, respectively. Therefore, different acids with higher dissociation constants were tested to promote the ionization of LIN and STD in a non-aqueous condition. Trifluoroacetic acid allowed for protonated basic nitrogen atoms of both of the weak bases of the oxazolidinone compounds. Interestingly, the difference in one atom (more electronegative) of sulfur instead of oxygen means that STD is a stronger base than LIN, which results in the stronger electrostatic interactions of STD with the tested CD than the interactions of LIN with the chiral selector. Based on the effective mobility measurements at different concentrations of HDAS-β-CD, it was revealed that STD and LIN are weakly bound to HDAS-β-CD because they remained cationic throughout the entire concentration range tested, bearing in mind that the CD used has a negative charge over a wide pH range. However, STD interacts more strongly with HDAS-β-CD than LIN, behaving according to the rule. In addition, STD interacting with HDAS-β-CD required a lower concentration of chiral selector in order to differentiate between the velocity of transient diastereoisomeric complexes than interacting with HDMS-β-CD. However, (*R*)-STD migrated after the main peak in the case of HDAS-β-CD, whereas a reversal of the EMO for HDMS-β-CD was observed (see Table 1). Hence, in this case, the reversal of the enantiomer affinity pattern depends on the substituents at position 2 and 3 of the HDAS-β-CD and HDMS-β-CD from acetyl to methyl group, respectively.

Although the STD has a structure similar to LIN, it nevertheless has low water solubility and therefore was selected. Therefore, it was decided that NMR measurements, which aimed to examine the interaction of STD with HDAS-β-CD, would be carried out in CD_3_OD and in CD_3_OD acidified with DCl, i.e., in solutions simulating the state where the enantiomeric STD separation was performed by NACE.

The ^1^H NMR spectrum of STD in CD_3_OD acidified with DCl was similar to that in CD_3_OD, but chemical shift changes of proton signals were observed, which confirms the protonation of the nitrogen atom of the thiomorpholine ring in STD. Analysis of the ^1^H NMR spectrum of STD and HDAS-β-CD mixture in CD_3_OD indicates that the complex between species has not been created or is very weak. The results of the ROESY and PFGSE measurements also confirmed this suggestion. On the other hand, for the mixture of STD and HDAS-β-CD dissolved in CD_3_OD acidified with DCl, the occurrence of some interactions was detected between STD and HDAS-β-CD in which aromatic and thiomorpholine rings of STD are involved. The diffusion measurements confirmed this conclusion.

## 2. Results and Discussion

The most likely mean geometries of the supramolecular complexes studied and their dynamics (changes in geometry over time) were determined using molecular dynamics methods. Enthalpy values of complex binding were calculated by long-term simulations of molecular dynamics in water and by MM/GBSA and MM/PBSA methods. The binding energy, or generally binding affinity, is an important metric in this type of research. A wide range of computational techniques exists to estimate the binding affinity of host–guest complexes, including statistical regression, knowledge-based neural networks, molecular mechanics-based free energy calculation, and free energy perturbation. In this work, the free energy type of calculations are conducted. For free energy or enthalpy calculation to be reliable, a proper representation of the physical system, an accurate force field, and an adequate sampling of the relevant molecular configurations must exist. In the case of CD complexes, the last remark seems to be particularly important. HDAS-β-CD, similar to other CDs, certainly exhibits high configurational dynamics, and for proper sampling of the energy surface, an excessive amount of molecular dynamics simulation steps is needed. Fortunately, the use of software powered by GPU graphics cards has opened up new horizons for large simulations. The results of molecular dynamics calculations for many configurations of oxazolidinones complexes with HDAS-β-CD, each lasting 20 µs, are presented.

The fundamental issue in enantioselective computer modeling is the concept of the molecular potential energy surface, which determines the dynamic features and shape of the interacting molecules. This raises two basic questions, namely, where to locate the selectand relative to the selector and how many selector–selectand complexes with all possible reciprocal orientations must be computed to adequately represent the system [14]. To reduce the number of samplings on the potential energy surface and docking, molecular dynamics or Monte Carlo methods are mainly used. These methods provide a better understanding, at the molecular level, of the structure and dynamics of enantioseparation processes [40].

**Figure 2:**
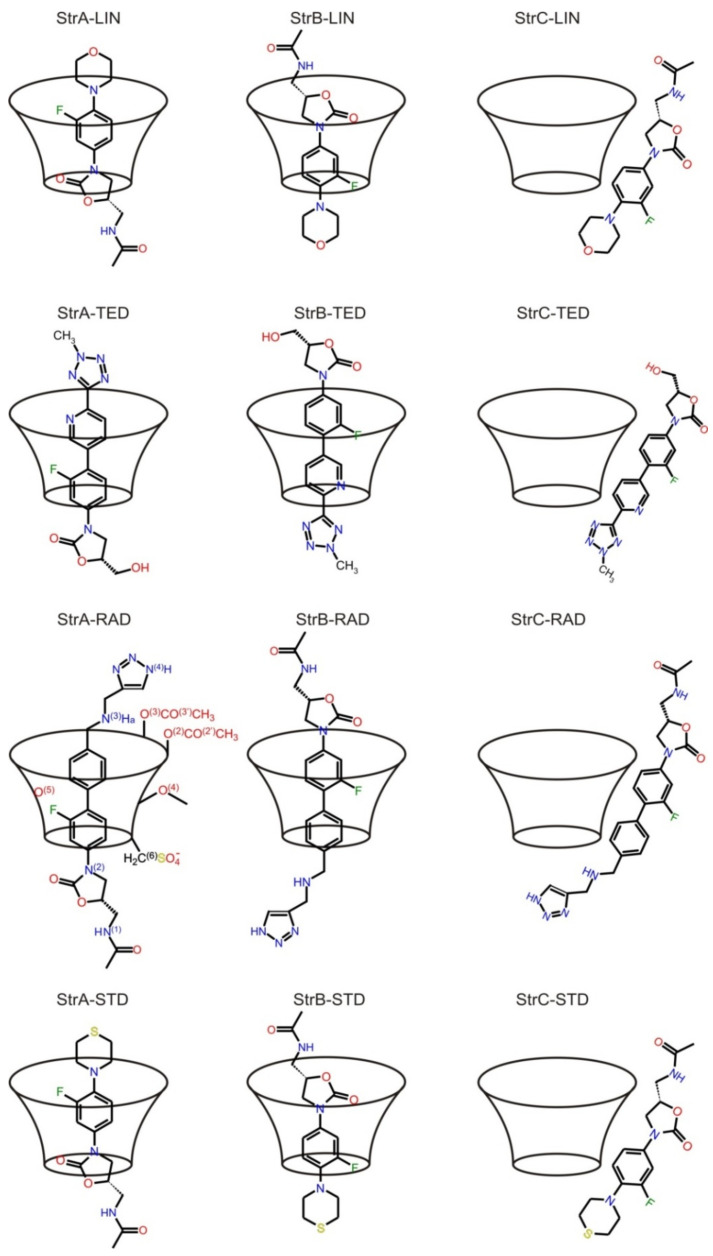
Schematic representation of TED, RAD, SUT [12], and LIN [5] complexes with HDAS-β-CD.

**Figure 3:**
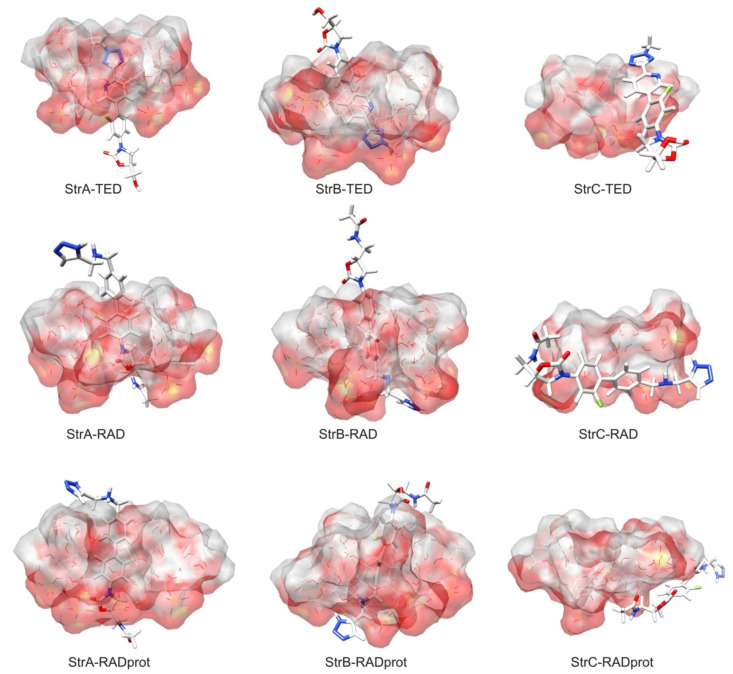
Representative conformations from the most probable cluster based on RMSD of the TED and RAD complexes with HDAS-β-CD.

The TED–HDAS-β-CD and RAD–HDAS-β-CD complexes were selected for the study, and they were theoretically investigated using molecular dynamic (MD) simulations and free energy calculations. The goal was to gain a better understanding of the structure and dynamic properties of inclusion of TED and RAD in the CD cavity compared to STD [12] and LIN [5], for which the results have already been published. These results constitute new and valuable information on the TED and RAD binding strength, the key guest–host interaction, and the binding free energy of complex formation. Schematic representations of the studied complexes are shown in Figure 2, while the geometries of all calculated complexes are shown in Figure 3.

The results of the energy calculations for complexes of oxazolidinones with HDAS-β-CD are presented in Table 2. The entropic contributions were neglected in the calculations, so only enthalpies were compared. This approximation was used because the current methods of calculating entropy are very imprecise and computationally demanding [41]. Generally, only similar structures that probably have very similar entropies were compared. The MD stands for the calculations made by the explicit water molecular dynamics simulations, and PBSA (GBSA) for the Poisson Boltzmann or the Generalized Born surface area methods. For comparison, Table 2 also presents the previously published energies calculated with the same methods for STD [12] and a slightly different method for LIN [6]. Additionally, the experimental values of binding constants also previously determined by various methods are summarized in Table 1. By comparing these two tables, it can be confirmed that the calculated enthalpies quite reliably reflect at least the order of changes in the values of the experimental binding constants. However, a more detailed analysis is difficult because both the experimental binding constants and the calculated absolute values of the binding energies may be subject to large inaccuracies. In the latter case, an accuracy better than 1–2 kcal/mol should not be expected, even with the most advanced calculation methods [42].

**Figure 4:**
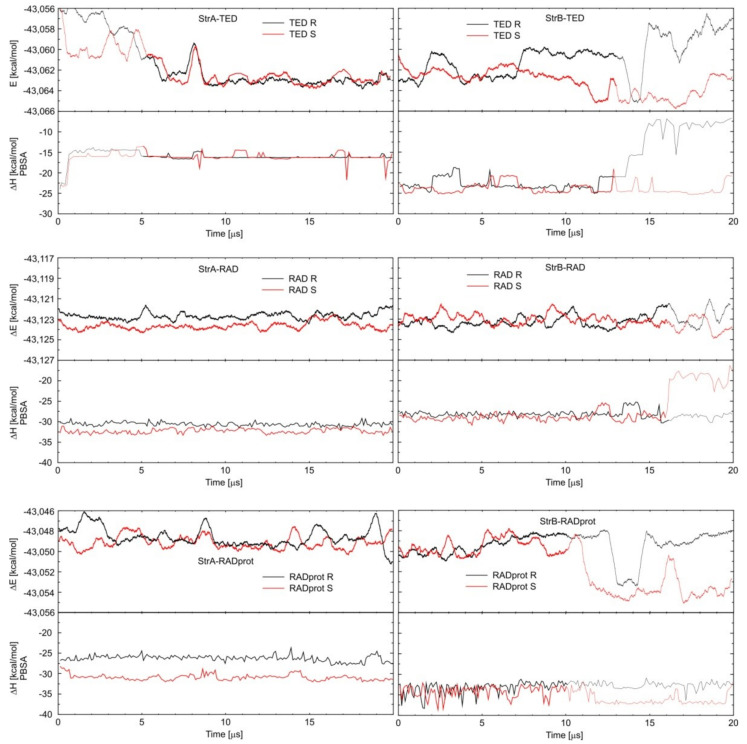
Energy changes during molecular dynamics simulations. The simulation ranges from which the average energies were calculated are shown in bold in Table 2.

**Figure 5:**
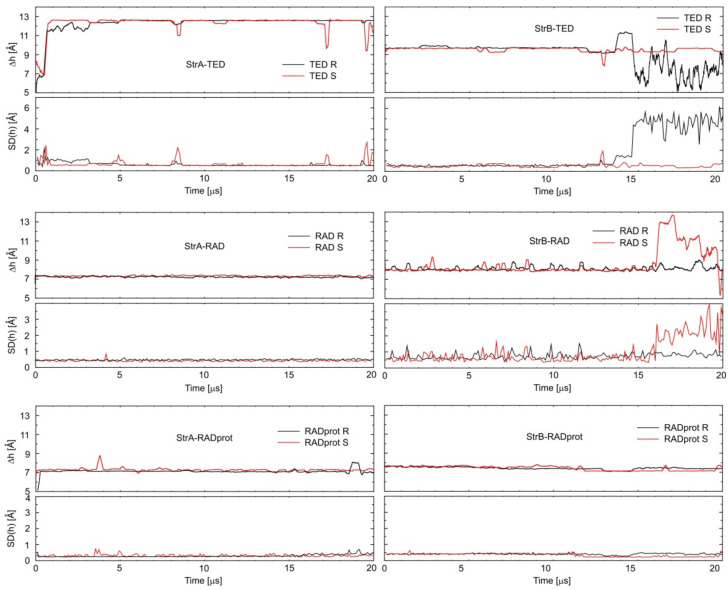
Changes in the distances Δh (and its standard deviations SD(h)) between the asymmetric carbon atom of oxazolidinone and the plane intersecting the centers of the cyclodextrin sugar rings during MD calculations.

In the case of the experimental results, it is easy to notice that when changing the measurement method or its conditions for LIN, very large changes in the K_a_ value are observed. Changing the calculation method also leads to large differences in the obtained absolute energy values. Fortunately, for both methods, it seems much more reliable to compare the relative values, e.g., only between different conformations or enantiomers of a given complex. It is clear from the calculations that TED and RAD binds preferably inside the CD cavity. The binding outside is unlikely, just like for STD and LIN. However, during a sufficiently long molecular dynamics simulation for one of the StrB-TED-(*R*) isomers, an escape from the cavity was observed, and TED began to bind outside the HDAS-β-CD. During this process, a rapid increase in energy was observed, starting from about 14 µs of the MD simulation; see Figure 4. In fact, after that time, the StrB structure was transformed into StrC, and the binding energies became comparable (StrB and StrC). From the energetic point of view, the opposite process should also be observed; i.e., the migration of TED back to the interior of the CD cavity. Unfortunately, even 20 µs of MD simulations seems far too short to observe the full cycle of complexation and decomplexation of oxazolidinones with HDAS-β-CD. A similar process can also be observed for the StrB-RAD-(*S*). However, in this case, only significant protruding of the RAD molecule from inside the CD was observed after approximately 16 µs of the MD simulation. Only the triazole ring was still immersed in the CD cavity, and the binding energy increased slightly. The process is well illustrated in Figure 5, which shows that the depth of CD penetration by the StrB-RAD-(*S*) molecule decreases after 16 µs of the simulation. For StrB-TED-(*R*) in the same figure, it can be seen that the penetration depth is increasing; however, this is a random coincidence, as we are actually measuring the distance between the chiral carbon atom of the TED and the plane through the centers of the HDAS-β-CD sugar rings, not only the distance to the center of the HDAS-β-CD. Interestingly, at the same time, the standard deviation of this distance increases significantly. This indicates that the binding of the TED to the exterior of CD is much less specific. The opposite effect can be observed for the protonated RAD complex (StrB-RADprot-(*S*)). After 12 µs of the simulation, a significant decrease in the value of the standard deviation can be observed, while the value of the same penetration depth was only slightly changed. At the same time, a decrease in energy can be observed (increase in the binding strength), as seen in Figure 4. This effect can be attributed to the formation of a new intermolecular hydrogen bond to stabilize the complex. The results are summarized in Table 3.

Generally, protonated RAD forms the strongest complexes (Table 2) and binds deep within the CD cavity. It can be assumed that hydrogen bonds are largely responsible for this effect. This is evidenced by the fact that a direct correlation can be found between the number of hydrogen bonds and the bond strength. The non-protonated RAD complexes, with a slightly fewer number of hydrogen bonds, also have slightly lower binding energies than protonated RAD. TED complexes, which have the lowest binding energies, do not form hydrogen bonds at all (Table 2). The calculated binding strengths decrease gradually in the order: RAD-prot, STD-prot, RAD, LIN, STD, and TED. For almost all oxazolidinones, the most preferred conformations are those in which the oxazolidinone moiety protrudes from a wide part of the CD cavity (StrB). The exception is unprotonated RAD, for which both orientations of the molecule in the CD cavity are almost equally likely.

Changing the solvent from water to MeOH/ACN (80/20 *v/v*) (mixture used in NACE experiments) only slightly increases the binding strength of the complexes (Table 2).

An attempt was made to determine the enantioselectivity of oxazolidinone–HDAS-β-CD complex formations. As the expected differences in the energies of the enantiomeric complexes were generally small, a very careful approach to the analysis of the calculation results was required. The main problem seems to be that the observed complexation processes during even MD simulations as long as 20 µs were not fully reversible and equilibrated. As mentioned earlier, quite a few anomalies in energy fluctuations related to equilibration, decomplexation, or hydrogen bonding have been observed. To try to eliminate these anomalies, only selected time intervals (without anomalies) of the molecular dynamics simulation were used to calculate the average energies in Table 2. These selections are marked in bold in Figure 4. By comparing the energies obtained in pairs of enantiomers with each other, the preferred complex can be identified. The lowest energy complexes, marked in bold in Table 2, should be considered as dominant in the solutions, and thus, they are mainly responsible for the experimentally observed enantiodiscrimination.

In conclusion, (*S*)-oxazolidinone enantiomeric complexes appear to be preferred for TED and non-protonated RAD, which is fully in line with the experimental data in Table 1. In the case of protonated RAD, the conclusions are less clear-cut. The differences in energies are smaller, and the preferred enantiomer depends on the calculation method. This is probably due to the fact that the StrB–RADprot complex was the most poorly equilibrated during the calculations, and its average energies were strongly overestimated, as can be clearly seen in Figure 4. In addition to the problems with equilibrating, it is worth asking if the molecular mechanics-based MD computations are generally accurate enough to notice the enantioselectivity of the complexation and if the observed energy differences are not just random fluctuations. Many examples of successful molecular modeling in cases of enantiodiscrimination or enantioseparation by cyclodextrines can be found in the literature [19,43,44]. The calculated binding energies are certainly not quantitative, as the accuracy of the molecular mechanics calculations is rather limited [42]. However, it seems that qualitative analysis is possible. In Figure 5, it can be seen that in the case of the StrA–TED complex, the distance of the TED chiral carbon atom from the plane crossing the center of the cyclodextrin is by far the greatest. As a result, this part of the TED molecule extends significantly beyond the cyclodextrin molecule and cannot interact with it. So, no enantioselectivity in binding energies should be expected, and indeed, the calculations confirm this exactly.

## 3. Computational Methods

### 3.1. Preparing the Molecular Dynamic Calculations for TED and RAD

A few starting structures of TED and RAD (neutral for TED, and both neutral and protonated on the N3 nitrogen atom for RAD, as shown in Figure 2) were manually created and fully optimized with the density functional theory (DFT) method using 6-31G* basis set and B3LYP functional in the Gaussian 09 program [45].

Four of the lowest energy neutral TED structures were selected and used to calculate the restrained electrostatic potential (RESP) charges required in molecular dynamic (MD) simulations. The same procedure was repeated for both neutral and protonated RAD. The modified HDAS-β-CD was created by adding acetyl and sulfate groups to the native β-CD and fully optimizing the geometry with the same method as for TED and RAD, except that only one structure was used in the RESP calculations. The electrostatic potential (ESP) charges were obtained for all TED, RAD, and HDAS-β-CD molecules by the HF/6-31G* calculations using Gaussian 09. Next, the RESP charges were calculated by the charge fitting procedure of the antechamber module implemented in the Amber 15 suite of programs [46]. The missing general amber force field (GAFF) parameters were obtained using the parmchk module from Amber 15. Next, inclusion complexes with HDAS-β-CD were constructed for TED and RAD molecules for the (*R*)- and (*S*)- isomers. Six complexes with oxazolidinone rings were penetrating the narrow cavity of HDAS-β-CD, which were marked as StrA-TED-(*R*,*S*), StrA-RAD-(*R*,*S*), and StrB-RADprot-(*R*,*S*). Six complexes with opposite orientations with tetrazole (TED) or triazole (RAD) rings were penetrating the narrow cavity of HDAS-β-CD, which were marked as StrB-TED-(*R*,*S*), StrB-RAD-(*R*,*S*), and Str-RADprot-(*R*,*S*). Additionally, three non-inclusion complexes were constructed with TED and RAD molecules (with only *R* configuration on asymmetric carbon atoms) placed perpendicular to the HDAS-β-CD (StrC-TED-(*R*), StrC-RAD-(*R*), and StrC-RADprot-(*R*)). Each complex was neutralized by the addition of Na^+^ cations and then solvated by TIP3 water molecules with a spacing distance of about 14 Å around the system surface, forming a periodic box. The number of water molecules was adjusted to exactly 4509. The same neutralization and solvation procedure was repeated for HDAS-β-CD alone, TED-(*R*) and RAD-(*R*) (neutral and protonated), a pure box of 4509 TIP3 water molecules, and finally, a box of 4509 TIP3 water molecules with one NaCl molecule. All twenty-one systems were subjected to molecular dynamics simulations (MD) using pmemd.cuda Amber 14 module with the NVIDIA GPU acceleration. The Particle-Mesh Ewald (PME) method was used for the treatment of long-range electrostatic interactions with 9 Å cut-off for the unbounded Lennard–Jones interactions. The SHAKE algorithm was applied to constrain all bonds involving hydrogen atoms, and a 2fs time step was used in the dynamics simulation. First, the systems were minimized in two stages. The first stage restrains the atomic positions of the solute and only relaxes the water, and the second stage releases the restraint and allows all the atoms to relax (both with 10,000 steps of minimization). Next, the systems were slowly heated to 300 K using constant volume and temperature (NVT) ensemble and 1,000,000 steps with the Langevin dynamics for temperature control (gamma_ln = 1.0). Then, the systems were carefully equilibrated at constant pressure and temperature (NPT) ensemble simulations at 1 bar pressure with gamma_ln = 5.0. In the first stage, equilibrations were maintained until the system reached a convergent density value, usually for 50–100 ns. In the second stage, MD simulations were carried out for 500 ns, out of which was determined the average value of the system volumes used in subsequent simulations. Finally, the NVT molecular dynamics production runs were performed for 20 µs of simulations for each of the twenty-one structures.

### 3.2. Calculating Binding Enthalpies Using Explicit Water Molecular Dynamics Simulations (Absolute Binding Free Energy)

The binding enthalpies were computed generally following the web-based tutorial on the Amber home page [47] and the corresponding publication [48]. The binding enthalpies are defined as follows:

ΔH = <H>_complex_ + <H>_pure water_ − <H>_receptor_ − <H>_ligand_ (1)

where <H>_complex_, <H>_pure water_, <H>_receptor_, and <H>_ligand_ are the Boltzmann averaged total potential energies for the complex, pure water, receptor (HDAS-β-CD), and ligand (TED or RAD) simulations, respectively. For complexes with protonated RAD, the pure water was replaced by water with NaCl because the total number of all particles in the system must be constant. The energies were the mean values from up to 20 µs molecular dynamic simulations. Such long simulations were necessary in this method in order to be sure that the systems were sufficiently sampled. The reported errors were estimated from the comparison of partial energy averages for subsequent 1 µs runs over each MD trajectory. We believe that errors determined in this way reflect reality better than, for example, with blocking analysis types of errors.

### 3.3. Calculating Binding Free Energies (Enthalpies) Using MM-PBSA and MM-GBSA Methods

The binding free energies can also be calculated [49] by neglecting the explicit solvent molecules by dividing up the calculations according to the following equations:

ΔG°_Bind, Solv_ = ΔG°_Bind, Vacuum_ + ΔG°_Solv, Complex_ − (ΔG°_Solv, Ligand_ + ΔG°_Solv, Receptor_). (2)

Solvation free energies were calculated by either solving the linearized Poisson–Boltzmann or the Generalized Born equation for each of the three states (this gives the electrostatic contribution to the solvation free energy) and adding an empirical term for hydrophobic contributions:

ΔG°_Solv_ = ΔG°_Electrostatic,__ϵ=80_ − ΔG°_Electrostatic,__ϵ=1_ + ΔG°_Hydrophobic_. (3)

ΔG°_Vacuum_ was obtained by calculating the average interaction energy between the receptor and ligand and taking the entropy change upon binding into account, according to the following equation:

ΔG°_Vacuum_ = ΔE°_MM_ − TΔS° (4)

where G°_Bind, Solv_ is the free energy of binding of solvated molecules, G°_Bind, Vacuum_ is the binding free energy in vacuum, G°_Solv, Complex_, G°_Solv, Ligand_, and G°_Solv, Receptor_ are the solvation free energies for complex, ligand, and receptor molecules, respectively, G°_Electrostatic_ is the electrostatic solvation free energy, G°_Hydrophobic_ is the hydrophobic (non-polar) solvation free energy, E°_MM_ is the molecular mechanic energy, T is the temperature, S° is the entropy, and ϵ is the dielectric constant (80 for water and 1 for vacuum).

The contribution of entropy in our calculations has been neglected, as states with similar structures are compared, and therefore, most likely have similar entropies. Calculations were made for structures that regularly accumulated during up to the 20 µs of MD simulation runs, and up to the 1,000,000 structures were used for each system. The free energies of solvation are usually calculated for an aqueous solvent using a dielectric constant equal to 80. Calculations were also performed with the dielectric constant set at 33.7 in order to mimic the MeOH/ACN (80:20 *v/v*) solvent mixture used in the NACE experiments.

## 4. Conclusions

The chiral cEKC, combined with state-of-the-art instrumental NMR methods and computation methodologies, provided impulses to better understand the non-covalent intermolecular interactions of oxazolidinones with HDAS-β-CD.

Both cEKC experimental data and calculations show that RAD forms the strongest complexes with HDAS-β-CD, especially at low pH, when the nitrogen atom in the methylaminomethyl link is protonated. The increased binding strength of RAD to HDAS-β-CD can be explained by the formation of intermolecular hydrogen bonds not present for other oxazolidinones. Protonated RAD enters the CD molecule with a triazole part of the molecule, while at a neutral pH, it seems, with equal probability, that RAD may enter the cavity of the CD through both ends of the molecule. The binding strength of RAD to CD is so great that during cEKC experiments, the binding of RAD to the second CD molecule (1:2 stoichiometry) is also observed. Then, RAD is probably complexed at both ends. Of course, the binding strength of this second CD molecule is much smaller than the first (almost two orders of magnitude); therefore, it certainly has little effect on the enantioseparation mechanism. 

STD forms complexes with intermediate stability; however, similar to RAD, complexes are more stable at low pH. For STD, the conformation is always preferred when the thiomorpholine ring enters the inner of the CD. LIN, similar to STD, and RAD form a complex more stable at low pH. Calculations suggest that TED forms the weakest complex, entering the cavity of CD by the tetrazole part of the molecule. However, the experimentally determined values of the binding constant of TED with CD seem to indicate that TED should bind more tightly. However, a retrospective analysis of these data as well as the described methodological difficulties indicate that the given values were overestimated. This is confirmed by the fact that the measurement for LIN performed under the same conditions differs significantly from the previous ones.

Using computer modeling, it was possible to qualitatively confirm the difference in the strength of binding of individual optical isomers of oxazolidinones to HDAS-β-CD and, thus, predict the enantioseparation.

## Figures and Tables

**Table 1:** Summary of the fundamental parameters obtained in the cEKC studies and experimental values of binding constants for HDAS-β-CD.

Analyte	Type of BGE	Separation Conditions	EMO Reversal for a Leading Compound	Ref.	Method of Determination of Binding Constant	pH	Binding Constant Ka * [M^−1^]		Ref.
							-(*R)*	-(*S*)	
LIN leading	ACE	27.5 mM HDAS-β-CD in 50 mM borate buffer, pH 9.0, 15 °C, capillary (1), NP, 15 kV	18.75 mM HDAS-β-CD in 50 mM borate buffer, pH 8.0, 15 °C, capillary (2), RP, 15 kV	[4]	NMR diffusion, D_2_O buffer	2.4	**76 ± 5**	66 ± 5	[5]
						7.0	**52 ± 2**	35 ± 2	
LIN	ACE	37.5 mM HDAS-β-CD in 50 mM formic buffer, pH 4.0 with ACN (81.4:18.6, *v/v*), 27 °C, capillary (1), 12 kV, NP/ or 35 mM HDAS-β-CD in 50 mM borate buffer, pH 9.0 with ACN (81.4:18.6, *v/v*), 27 °C, capillary (1), 12 kV, NP	not observed	[6]	cEKC	4.0	1311 ± 256	**1507 ± 307**	[6]
						9.0	571 ± 80	**625 ± 88**	
TED leading						4.0	**747 ± 155**	728 ± 145	
						9.0	319 ± 51	**326 ± 50**	
TED		-	-	-	NMR diffusion, D_2_O	-	140 *±* 30	-	[8]
TED-PO_4_						-	98 ± 20	-	
LIN	ACE	6 mM HDAS-β-CD in 50 mM phosphoric buffer, pH 2.5, capillary (1), 28 kV, 17–27 °C, NP	18 mM HDAS-β-CD in 50 mM phosphate buffer, pH 6.6, 27 °C, capillary (1), NP	[9]	cEKC	2.5	41.5 ± 10	**42.9 ± 10**	[9]
						6.6	45.8 ± 10	**53.4 ± 10**	
RAD leading		20 mM HDAS-β-CD in 50 mM phosphoric buffer, pH 2.5, capillary (1), 28 kV, 52 °C, RP (partial resolution)				2.5	**2940 ± 600**	2817 ± 600	
						6.6	999 ± 200	**1082 ± 200**	
LIN	NACE	not observed with HDAS-β-CD	45 mM HDMS-β-CD in MeOH/ACN (85:15, *v/v*), 200 mM TFA/20 mM ammonium formate, capillary (1); 25 kV, 22 °C, NP	[12] nd		nd	nd	nd	-
STD leading		5 mM HDAS-β-CD in MeOH/ACN (85:15, *v/v*), 200 mM TFA/20 mM ammonium formate, capillary (1); 25 kV, 22 °C, NP							

Capillary (1)—uncoated fused silica capillary, capillary (2) neutral-coated capillary—zero EOF flow, NP—normal polarity, RP—reversed polarity, nd—not detected, * for the cEKC method, Ka is the apparent binding constant since the viscosity and the exact ionic strength of the buffer, which is affected by the increase in charged CD concentration, were not included in the calculations. The highest binding values are shown in bold.

**Table 2:** Calculated binding average energies (enthalpies) for oxazolidinones complexes with HDAS-β-CD. Averages are calculated only for the time intervals marked in bold in Figure 4.

Compound	Average Binding Enthalpies [kcal/mol] ^(b)^							
	StrA			StrB			StrC	
	H_2_O		MeOH/ACN	H_2_O		MeOH/ACN	H_2_O	
	MD	PBSA (GBSA)	PBSA (GBSA)	MD	PBSA (GBSA)	PBSA (GBSA)	MD	PBSA (GBSA)
LIN-(*R*) ^(a)^	−	−21.6 ± 4.8 (−21.1 ± 3.9)	−	−	−26.4 ± 4.5 (−31.7 ± 4.5)	-	-	-
LIN-S ^(a)^	−	−21.7 ± 5.0 (−22.5 ± 3.3)	−	−	24.7 ± 3.8 (−30.4 ± 2.6)	-	-	-
TED-(*R*)	−21.3 ± 1.7	−16.2 ± 0.2 (−18.8 ± 0.2)	−16.4 ± 0.2 (−19.1 ± 0.2)	−21.4 ± 1.2	−22.9 ± 1.0 (−27.9 ± 0.8)	−23.2 ± 1.1 (−28.2 ± 0.8)	−17.5 ± 2.2	−10.6 ± 2.8 (−9.9 ± 2.7)
TED-(*S*)	−21.4 ± 1.5	−16.1 ± 0.4 (−18.8 ± 0.4)	−16.4 ± 0.4 (−19.2 ± 0.4)	**−21.9 ± 0.5**	**−23.7 ± 1.1** **(−28.3 ± 1.0)**	**−24.0 ± 1.1** **(−28.6 ± 1.0)**	-	-
RAD-(*R*)	−23.4 ± 0.3	−28.6 ± 0.3 (−31.1 ± 0.3)	−29.3 ± 0.2 (−31.8 ± 0.3)	−23.6 ± 0.8	−28.2 ± 0.7 (−31.1 ± 0.7)	−29.1 ± 0.7 (−31.9 ± 0.7)	−21.9 ± 1.9	−17.2 ± 2.2 (−17.4 ± 2.9)
RAD-(*S*)	**−24.3 ± 0.4**	**−30.2 ± 0.4** **(−32.3 ± 0.5)**	−31.2 ± 0.4 (−33.2 ± 0.5)	−23.8 ± 0.6	27.0 ± 3.8 (−29.9 ± 4.1)	27.8 ± 4.1 (−30.5 ± 4.1)	-	-
RADprot-(*R*)	−26.6 ± 0.9	−26.6 ± 0.5 (−30.4 ± 0.4)	−30.8 ± 0.7 (−33.5± 0.3)	**−27.7 ± 0.7**	−33.3 ± 0.8 (−36.1 ± 0.7)	−40.6 ± 0.8 (−40.8± 0.7)	−20.2 ± 3.2	−16.4 ± 1.8 −15.8 ± 2.2
RADprot-(*S*)	−27.1 ± 0.6	−31.3 ± 0.5 (−34.4 ± 0.3)	−35.8 ± 0.7 (−37.6 ± 0.4)	−27.5 ± 0.8	**−34.2 ± 0.7** **(−36.9 ± 0.5)**	−41.3 ± 0.8 (−41.5 ± 0.6)	-	-
STD ^(a)^	−18.9 ± 0.7	−23.4 ± 0.7 (−25.9 ± 0.8)	−24.1 ± 0.7 (−26.6 ± 0.7)	−20.2 ± 0.8	−25.8 ± 2.1 (−29.3 ± 1.0)	−26.6 ± 2.1 (−29.9 ± 1.0)	−16.5 ± 1.1	−5.5 ± 4.0 −5.5 ± 3.5
STDprot ^(a)^	−18.8 ± 0.7	−23.8 ± 0.9 (−26.1 ± 0.8)	−24.3 ± 1.2 (−26.7 ± 1.1)	−19.9 ± 0.6	−26.2 ± 1.0 (−32.1 ± 0.9)	−32.8 ± 1.1 (−36.2 ± 1.2)	-	-

^(a)^ Calculation results taken from the previously published work for STD [12] and for LIN [5]. ^(b)^ The lowest energies for oxazolidinones complexes are shown in bold.

**Table 3:** Intermolecular hydrogen bonds (H-bonds) in oxazolidinone–HDAS-β-CD complexes.

Complex Structure	Intermolecular H-Bonds			
	Name	Av. Distance [Å]	Population	Av. H-Bonds No.
StrA–TED	-	-	No	0
StrB–TED	-	-	No	0
StrA–RAD	RAD-N^(1)^**H** … CD-S**O**_4_^−^	2.29 +/− 0.47	High	1.30 (*R*) 2.06 (*S*)
	RAD-N^(3)^**H**a … CD-**O**3′	2.24 +/− 0.34	High	
	RAD-N^(4)^**H** … CD-**O**3′	2.17 +/− 0.30	High	
	RAD-N^(4)^**H** … CD-**O**2′	2.29 +/− 0.34	Mid	
	RAD-N^(3)^**H**a … CD-**O**3	2.60 +/− 0.28	Mid	
	RAD-N^(3)^**H**a … CD-**O**2	2.57 +/− 0.31	Mid	
	RAD-N^(3)^**H**a … CD-**O**2′	2.75 +/− 0.30	Low	
StrB–RAD	RAD-N^(4)^**H** … CD-S**O**_4_^−^	2.24 +/− 0.35	High	2.08 (*R*) 2.01 (*S*)
	RAD-N^(3)^**H**a … CD-S**O**_4_^−^	2.30 +/− 0.35	High	
	RAD-N^(3)^**H**a … CD-**O**5	2.44 +/− 0.31	Mid	
	RAD-N^(4)^**H** … CD-**O**5	2.39 +/− 0.31	Mid	
	RAD-N^(4)^**H** … CD-**O**6	2.44 +/− 0.29	Low	
StrA–RADprot	RADprot-N^(1)^**H** … CD-S**O**_4_^−^	2.29 +/− 0.46	High	1.94 (*R*) 2.59 (*S*)
	RADprot-N^(3)^**H**b … CD-**O**2′	2.12 +/− 0.28	High	
	RADprot-N^(3)^**H**b … CD-**O**3′	2.18 +/− 0.35	High	
	RADprot-N^(3)^**H**a … CD-**O**2′	2.14 +/− 0.29	High	
	RADprot-N^(3)^**H**a … CD-**O**3′	2.08 +/− 0.23	High	
	RADprot-N^(4)^**H** … CD-**O**2′	2.12 +/− 0.28	Mid	
	RADprot-N^(4)^**H** … CD-**O**3′	2.22 +/− 0.32	Mid	
StrB–RADprot	RADprot-N^(3)^**H**a … CD-S**O**_4_^−^	2.27 +/− 0.37	High	3.25 (*R*) 3.21 (*S*) ^(^*^)^ *3.65 (S)*
	RADprot-N^(3)^**H**b … CD-S**O**_4_^−^	2.23 +/− 0.35	High	
	RADprot-N^(4)^**H** … CD-S**O**_4_^−^	2.23 +/− 0.37	High	
	RADprot-N^(1)^**H** … CD-**O**2′	2.23 +/− 0.41	Low	
	^(^*^)^*RADprot-N^(1)^**H** … CD-**O**3′*	^(^*^)^ *2.54 +/− 0.44*	^(^*^)^*High*	

^(^*^)^ H-bond created in 12 µs of simulation for the StrB–RADprot(*S*) complex.

## Data Availability

Not applicable.
